# 

**Published:** 1897-01

**Authors:** 


ESTABLISHED 1855. SUBSCRIPTION, $2.00.

### ATLANTA MEDICAL AND SURGICAL JOURNAL.

VOLUME XIII. NEW SERIES. ATLANTA, GA., JANUARY, 1897. No. 11.

